# Yoga May Mitigate Decreases in High School Grades

**DOI:** 10.1155/2015/259814

**Published:** 2015-08-10

**Authors:** Bethany Butzer, Max van Over, Jessica J. Noggle Taylor, Sat Bir S. Khalsa

**Affiliations:** ^1^Department of Medicine, Brigham and Women's Hospital, Harvard Medical School, Boston, MA 02115, USA; ^2^Department of Public Health, University of Massachusetts, Amherst, MA 01003, USA

## Abstract

This study involves an exploratory examination of the effects of a 12-week school-based yoga intervention on changes in grade point average (GPA) in 9th and 10th grade students. Participants included 95 high school students who had registered for physical education (PE) in spring 2010. PE class sections were group randomized to receive either a yoga intervention or a PE-as-usual control condition. The yoga intervention took place during the entire third quarter and half of the fourth quarter of the school year, and quarterly GPA was collected via school records at the end of the school year. Results revealed a significant interaction between group and quarter suggesting that GPA differed between the yoga and control groups over time. Post hoc tests revealed that while both groups exhibited a general decline in GPA over the school year, the control group exhibited a significantly greater decline in GPA from quarter 1 to quarter 3 than the yoga group. Both groups showed equivalent declines in GPA in quarter 4 after the yoga intervention had ended. The results suggest that yoga may have a protective effect on academic performance by preventing declines in GPA; however these preventive effects may not persist once yoga practice is discontinued.

## 1. Introduction

Most educational settings use academic performance as a primary indicator of student success. Unfortunately, many students struggle to achieve and/or maintain adequate grades, which may result in detrimental outcomes such as academic disengagement and dropout [1]. While some school-based interventions, such as social-emotional learning programs, have shown promise with regard to improving academic performance [2], these programs are often difficult to integrate into the academic curriculum [3]. Thus it would be advantageous for schools to explore interventions that increase academic performance and are feasible to implement. One option that shows promise in this regard is mind-body practices such as yoga.

Yoga is a holistic system of mind-body practices that was originally developed as a practice for achieving optimal mental, emotional, and physical health and ultimately unitive states of consciousness. A growing body of research evidence suggests that yoga has significant psychophysiological benefits and clinical relevance as a therapeutic practice [4]. Yoga in its traditional form incorporates four primary components: physical postures/exercises to promote strength and flexibility, breathing exercises to enhance respiratory functioning, deep relaxation techniques to cultivate the ability to physically and mentally release tension, and meditation/mindfulness practices to enhance mind-body awareness and improve attention and emotion and stress regulation skills [5].

Two systematic review papers [6, 7] and one clinical review paper [8] suggest that yoga has beneficial effects on mental and physical health in children and adolescents. In addition, preliminary research has shown that school-based yoga interventions may improve several factors that are relevant to academic performance, such as emotional balance, attentional control, cognitive efficiency, and a number of positive psychosocial outcomes [9]. Research is also beginning to support the hypothesis that mind-body interventions may improve academic performance. For example, preliminary studies have shown that school-based mind-body interventions are related to improvements in performance impairment [10], English and mathematics scores [11], teacher ratings of academic performance [12], grade point average (GPA), work habits, cooperation [13], and tests of mathematics, science, and social studies [14]. Two preliminary studies of college students also suggest beneficial effects of mind-body practices on cumulative GPA [15] and cognitive performance [16]. Several qualitative studies have also found associations between school-based mind-body interventions and perceived improvements in restful alertness, ability to focus attention, keeping on task at school [17], as well as enhanced academic achievement [18], reductions in academic stress, improved attitudes toward school, and enhanced concentration [19].

While these preliminary studies suggest potential associations between school-based mind-body interventions and improvements in academic performance, this research suffers from several limitations such as low methodological quality and a reliance on single test scores and/or student/teacher self-report. Specifically, many prior studies that have examined the association between mind-body practices and academic performance have used self-report, single-arm trials in elementary schools. This is problematic considering that self-reports of grades may be less reliable than grades obtained from school records [20]. In addition, the majority of previous studies have examined meditation and/or mindfulness interventions, which, while being beneficial, focus almost exclusively on cognitive behavioral activity, specifically the control of attention. Yoga, on the other hand, includes additional components such as breath regulation and physical exercises and postures that have unique psychophysiological benefits that could be relevant to academic performance, such as stress reduction [21] and self-regulation [22]. To date, only two quantitative studies (described above) have examined the specific effects of yoga on academic performance [10, 14], and only one of these studies focused on high school students, suggesting a need for additional research in this area, particularly research using longer-term objective measures such as cumulative grade point average (GPA) in high school students.

The purpose of the present study is to report an exploratory analysis of the effects of a school-based yoga intervention on cumulative GPA obtained from high school records. To our knowledge, the current study represents the first examination of the effects of yoga on GPA in high school students.

## 2. Methods

### 2.1. Participants

Eligibility to participate in the study required registration for 9th or 10th grade physical education (PE) class at a public high school in western Massachusetts for the spring 2010 semester. During the 2009 and 2010 school year the school had 595 students registered from the 9th to 12th grade with a race and ethnicity composition of 91% white, 3.8% Hispanic, 3% multiracial or non-Hispanic, 1.7% African American, and 0.5% Asian. The graduation rate of the school in 2009-2010 was 91.2%, with 16.4% of the population considered of low income [23].

The only exclusion criteria were that the students must not have taken yoga in the previous semester and could not have any medical or psychological conditions that would prohibit yoga participation. Consent to participate in the yoga program and to complete self-report questionnaires was obtained using an opt-out, passive informed consent procedure. Specifically, informed consent letters were sent to the participants' parents explaining the details of the study. If the parents decided against their child's participation they conveyed their decision for opting their child out of the study to school or study staff. As per school policy, written parental consent was obtained specifically for the collection of student grades. All study procedures were approved by the Institutional Review Board at the Brigham and Women's Hospital.

### 2.2. Randomization and Data Collection

During the group randomization process, the eligible PE classes were randomly assigned to either receive the yoga intervention or continue as scheduled with PE-as-usual as the control condition. Three classes were assigned to the PE-as-usual condition and three classes were assigned to the yoga intervention, with a relatively equal amount of students in the intervention and control groups initially. At this particular school, the academic year was broken up into four 8-week quarters. Quarter 1 occurred from August to October, Quarter 2 occurred from November to January, Quarter 3 occurred from February to April, and Quarter 4 occurred from May to June. All student grade data were collected by the school at the end of each quarter and accessed by research study staff at the end of the school year. The yoga intervention began at the start of the third quarter and ended four weeks into the fourth quarter. Student demographic information was collected by study staff two weeks before the yoga intervention began.

### 2.3. Yoga Intervention

The yoga intervention was based on the Kripalu Yoga in the Schools (KYIS) curriculum developed by the Kripalu Center for Yoga and Health. The two yoga intervention instructors had completed 500 hours of yoga teacher training, including specialized training in teaching yoga to adolescents. The yoga instruction was designed to promote the engagement and compliance of students while mitigating any potential risk of injury. In each of the yoga classes there was one teaching assistant present who took attendance, kept notes for the teacher, and aided the students in performing the practices. The teaching assistants had completed 200 hours of yoga training. Additional care was also taken to ensure the yoga postures were adaptable for all fitness levels.

Participants in the three classrooms assigned to the yoga group practiced yoga two to three times per week for 12 weeks, instead of regularly scheduled PE sessions. The yoga intervention consisted of 28 instructed yoga sessions for two of the classrooms and 29 yoga sessions for the third classroom due to school scheduling differences. The 35- to 40-minute yoga sessions included 5 minutes of centering and breathing exercises, 5 minutes for an experiential game/activity, 5 minutes of warming up in basic yoga positions, 15 minutes of additional yoga postures, and 5 minutes of supine relaxation that included body scanning and breath awareness. Mindfulness and meditation practice were also included within the yoga curriculum. The themes of the teaching points that were taught during the classes pertained to yoga philosophy, the physiology of breathing, mind-body interactions, being present in the moment, and how yoga poses can affect stress, energy, and physiology.

### 2.4. Physical Education Control

Students randomized to the PE-as-usual control condition engaged in 35 to 40 minutes of the school's standard PE curriculum 2 to 3 times per week that lasted for the duration of the 12-week yoga intervention (28 total PE sessions). The participants that were registered for these classes dedicated 2 weeks to learning the history and rules of a sport as well as practicing the activity or sport. The 2-week units consisted of traditional sports such as tennis, volleyball, hockey, football, ultimate frisbee, and baseball. Nontraditional sports were also included in the PE curriculum. This included a ropes course, backcountry living skills, stress management, first aid/cardiac pulmonary resuscitation, planned parenthood, and health and wellness. Yoga practice and any information regarding yoga were not included in the standard PE curriculum.

### 2.5. Outcome Measures

In the present study, GPA was collected as a secondary outcome measure as part of a larger study designed to examine the effects of a school-based yoga intervention on psychosocial well-being. In other words, the grade data was obtained in a post hoc manner at the end of the school year, after all of the original study procedures, intervention, and outcome measures were complete. Outcome measures for the original study included self-report scales assessing a variety of psychosocial outcomes. However, based on the fact that the original study was not designed or sufficiently powered to examine both the psychosocial questionnaires and the GPA data in combination, the present paper focuses exclusively on an exploratory analysis of the GPA data. The quantitative results of the original study are unpublished; however the qualitative results are presented in Conboy et al. [19].

GPA is a commonly used method to measure student performance and is calculated by converting letter grades into grade points that take into account the number of credit hours attempted and relative difficulty of the class. At the end of the 2009-2010 academic year, the school provided study staff with participants' raw percentage grades for each quarter across the year. The Tennessee Uniform Grade Point Average standards were used to convert the raw percentage data to a weighted GPA, which takes into account a higher weighting for advanced classes. In particular, the school categorized advanced placement, honors, and college preparatory classes into the same weighted group. Based on the Tennessee Uniform Grade Point Average standards, 0.5 was added onto the GPA for these classes. Tennessee Uniform Grade Point Averages range from 4.00 for an “A” (93%–100%) to 0.00 for an “F” (0%–69%).

### 2.6. Data Analysis

Univariate ANOVAs were conducted to test for potential gender, grade, and classroom differences in baseline (first quarter) GPA. Parametric assumptions such as sphericity and normality were also examined. Due to the exploratory nature of this study and its small sample size, the primary analyses were conducted at the individual rather than classroom level. In order to test for potential differences in GPA over time between the yoga and PE-as-usual controls, a split-plot ANOVA was conducted in which condition (yoga; control) served as the between-subjects factor, and quarter (1; 2; 3; 4) served as the within-subjects factor. Three post hoc independent-samples *t*-tests were conducted to compare the yoga and control groups on the difference scores of baseline GPA subtracted from second, third, and fourth quarter GPA. The study design incorporated intention-to-treat principles, so that every participant distributed into a treatment group initially was viewed as being a part of that same group during the data analysis. Alpha was set at 0.05 for the primary split-plot ANOVA. In order to control for Type I error, a Bonferroni correction was used for the post hoc *t*-tests, which set alpha at 0.02 for these post hoc analyses (i.e., 0.05/3 = 0.02). Data was analyzed using SPSS version 22.

## 3. Results

### 3.1. Demographics

The six PE class sections that were eligible for participation in the present study had a cumulative sample size of 115 students. The class sizes ranged from 12 to 18 students. Three students were never assigned to a treatment condition due to opting out of the study (2 students) and dropping out of school (1 student) prior to random assignment. Fourteen participants who were assigned to the yoga condition were excluded from the analysis due to a lack of parental consent regarding the collection of grades data (7 students), dropping out of school (2 students), or opting out of the study (5 students). Three participants from the control group were excluded due to a lack of parental consent to collect grade data. The resulting sample size used in the analysis included 44 students in the yoga group and 51 students in the control group (95 students total). The sample was mostly comprised of 9th and 10th grade students (47 and 46 students, resp.), but there were a small number of 11th grade participants (2 students). Baseline demographics of the sample are shown in Table 1 and suggest that the sample was primarily comprised of white participants, with slightly more females than males.

### 3.2. Baseline Analyses

Assessments of normality revealed that the GPA data for all four quarters were negatively skewed; however this is to be expected for data pertaining to academic performance [24]. In particular, Micceri [24] reviewed 440 distributions from 46 academic tests in 89 different populations to determine whether the data was normally distributed. The results showed that none of the distributions passed all the tests of normality. Micceri also acknowledged the robustness of most parametric statistical tests in handling negative skew. Thus, the present study analyzed GPA without applying any data transformations.

Potential baseline differences in GPA based on classroom were examined by conducting a univariate ANOVA. The between-subjects factor of classroom was a significant contributor to 1st quarter GPA, *F*(5,89) = 3.66, *p* = 0.005. Tukey post hoc comparisons were used to determine which classrooms contributed to this effect. One of the classrooms was found to have a significantly lower mean baseline GPA (*M* = 2.66, SD = 0.88) than three other classrooms *M* = 3.57, SD = 0.37, *p* = 0.001; *M* = 3.34, SD = 0.42, *p* = 0.050; and *M* = 3.36, SD = 0.38, *p* = 0.025. No other classrooms significantly differed from one another.

Potential baseline differences in GPA relative to grade level (9th, 10th, or 11th) were examined using a univariate ANOVA, whereas the association between gender (male and female) and 1st quarter GPA was examined using independent-samples *t*-tests. Grade level was not found to be a statistically significant contributor to 1st quarter GPA, *F*(2,92) = 1.08, *p* = 0.344. In addition, males (*M* = 3.29, SD = 0.55) and females (*M* = 3.21, SD = 0.70) did not significantly differ on baseline GPA, *t*(93) = 0.65, *p* = 0.52.

### 3.3. Analysis of Change in GPA over Time by Group

**Figure 1:**
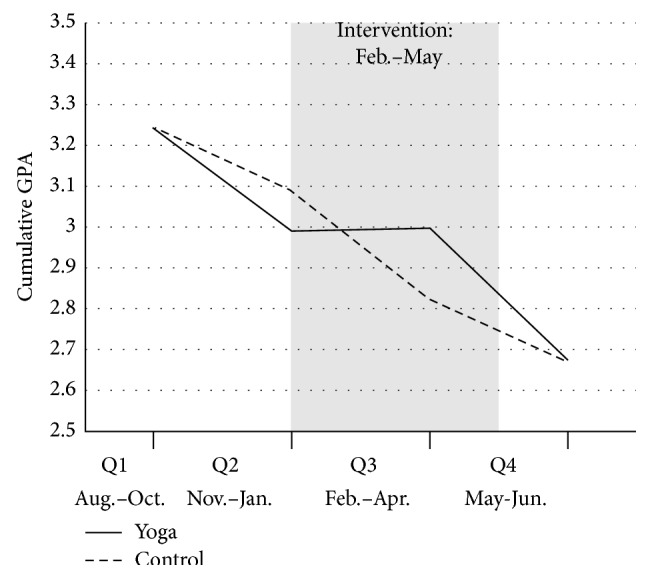
Mean cumulative GPA for the yoga and control groups over four academic quarters. Q1 = first quarter; Q2 = second quarter; Q3 = third quarter; Q4 = fourth quarter.

Means and standard deviations for cumulative GPA for each group at each quarter are presented in Table 2. A split-plot ANOVA was conducted to examine whether GPA differed between the yoga and control groups over time. Mauchly's test of sphericity yielded significant results; thus the Greenhouse-Geisser correction was used. The within-subject main effect of quarter was found to be significant, suggesting that GPA was changing over time for both groups *F*(2.41, 224.31) = 58.81, *p* = 0.000. In particular, mean GPA showed a decreasing trend over the four quarters for both groups. However, this main effect was qualified by significant interaction between group and quarter, suggesting that the pattern of change differed between the two groups over time, *F*(2.41, 224.31) = 3.43, *p* = 0.03. Specifically, the yoga group maintained approximately the same GPA from the second to third quarter, whereas the control group continued on a downward trend during this time period (Figure 1).

**Figure 2:**
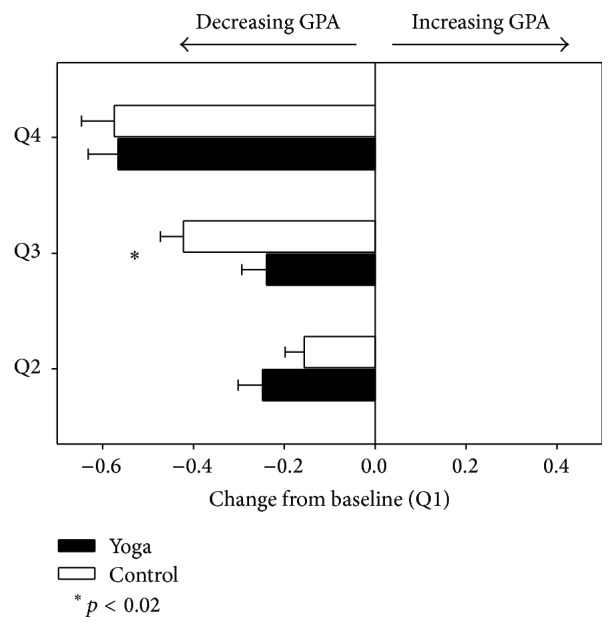
Difference scores between quarters 2, 3, and 4 relative to baseline (quarter 1) for the yoga and control groups. Q1 = first quarter; Q2 = second quarter; Q3 = third quarter; Q4 = fourth quarter.

In order to further probe this significant interaction, post hoc analyses were conducted to examine potential differences between the yoga and control groups with regard to changes in GPA relative to baseline. Difference scores were calculated by subtracting baseline GPA (quarter 1) from GPA at quarters 2, 3, and 4. Independent-samples *t*-tests on these difference scores revealed that while both groups showed equivalent declines in GPA from quarter 1 to quarter 2 and from quarter 1 to quarter 4 the control group showed a significantly greater decline in GPA from quarter 1 to quarter 3 (*M* = −0.42, SD = 0.36) than the yoga group (*M* = −0.24, SD = 0.37); *t*(93) = −2.46, *p* = 0.016 (Figure 2).

## 4. Discussion

The present findings suggest that participation in a school-based yoga intervention may have had a preventive effect by reducing the likelihood of declines in GPA over time. In particular, the results revealed a steeper downward trend in GPA in the control group compared to the yoga group during the third quarter, which is when the majority of the 12-week yoga intervention took place. This finding is consistent with qualitative interviews conducted with a subset of yoga students from the current study, in which some students reported decreased academic stress and better attitudes about school during the yoga program, although most students did not perceive a direct effect of yoga on their grades [19].

It is of interest to note that after the yoga intervention was complete, the mean GPA for the yoga group continued on a downward trend that was similar to the control group. This suggests that any protective effects of yoga on GPA may not persist once yoga practice is discontinued. To maintain GPA, it is possible that yoga may need to be practiced throughout the school year and/or may require yoga interventions of a longer duration (e.g., multiyear). To date, very few studies have examined the optimal frequency and duration of mind-body interventions in relation to academic performance [25]. An exception in this regard is a study by Benson et al, [13], in which middle school students underwent varying levels of a relaxation response curriculum that incorporated aspects of yoga. The results showed that students who took more than two semester-long classes with teachers trained in the relaxation response curriculum had significantly higher GPAs than students who had two or fewer exposures. This research suggests that there may be a dose-response relationship between mind-body practices and GPA. It is possible that the intervention that was used in the present study, which lasted only three quarters of a semester, may not have been of a long enough duration to promote continued maintenance of, or improvements in, GPA. Indeed, the mean GPA for the yoga and control groups did not differ at the end of the 4th quarter, despite the fact that students in the yoga group received yoga for half of this quarter (i.e., 4 weeks of the 8-week quarter). It is possible that students may need regular and persistent access to yoga, particularly during exam periods, in order for these types of programs to have an impact on GPA; however future research should explore this possibility more rigorously.

It is also important to note that both groups showed a general decline in GPA from the beginning to the end of the school year. This finding is similar to a large longitudinal study of college GPA across eight academic semesters, which revealed a pattern in which grades fell during the second semester, rose slightly, and then decreased again during the final academic term [26]. However there is a paucity of research on changes in high school GPA across a single school year, making it difficult to assess whether the results of the present study are consistent with national norms. Future research should examine these factors.

## 5. Potential Mechanisms

The current study raises questions regarding the potential mechanisms that may be responsible for the effects of yoga and meditation on academic performance. The use of a PE-as-usual control group allowed for an examination of potential differences between physical activity alone and yoga in relation to academic performance. Physical activity is one component of yoga and has been associated with improved academic performance in adolescents [27]. Yoga, however, involves additional elements including ethics, breathing exercises, and meditation techniques, which may provide unique benefits. The current findings suggest that yoga may be superior to physical education with regard to preventing declines in GPA over time; however due to the exploratory nature of this study it will be important for future research to further examine and replicate these results.

A broader possibility is that yoga may improve academic performance by enhancing emotional, cognitive, and behavioral self-regulation which may, in turn, mitigate stress, thus leading to enhanced attention and learning. Self-regulation refers to “efforts of monitoring, willpower, and motivation to manage or alter one's incipient responses and impulses so as to pursue or maintain explicit goals or standards” [28]. A growing body of research suggests that yoga interventions of relatively short durations (e.g., 8 to 12 weeks or less) may facilitate self-regulation in response to stress [28]. Thus it is possible that yoga-based improvements in self-regulatory processes might enhance high school students' ability to cope with stress, thus preventing stress-related declines in academic performance. High school students in the United States are under high levels of stress due to increased academic workloads, large classroom sizes, and receiving less individual attention from teachers [29]. High levels of stress at an early age can negatively impact brain development and are likely to have adverse effects on attention- and learning-related factors such as executive functioning and working memory, which may ultimately lead to poor academic performance [30]. Prior research suggests that yoga participation may improve several aspects of attention and learning, such as cognitive efficiency [9], spatial memory and executive functioning [31], time on task [32], and metacognition [33]. Qualitative results from students in the present study support the idea that yoga may mitigate academic-related stress [19], thereby improving academic performance. Yet without definitive studies on the relative influence of yoga on stress, cognition, and academic performance, it is also possible that yoga-based improvements in self-regulation may directly enhance students' cognitive abilities, which may have positive effects on academic skills.

A related possibility is that yoga's influence on the self-regulation of stress responses may improve general psychosocial well-being, which may be associated with improvements in academic performance. High school students are at risk for developing stress-related illnesses that may impact academics, including psychological disorders [29]. Indeed, poor academic performance [34] and higher rates of school dropout [35] have been linked to psychiatric disorders such as depression. Previous studies have shown that school-based yoga interventions, even interventions of relatively short duration (e.g., 8 to 12 weeks or less), may have beneficial effects on the mental health status of students [9]. For example, two of our studies examining a 10- and 11-week school-based yoga intervention showed that these interventions may prevent declines in psychosocial well-being [36, 37]. Thus, it could be the case that by enhancing self-regulation, school-based yoga programs may improve students' stress coping abilities, thereby benefiting both psychosocial well-being and academic performance in a yet-to-be-defined, possibly synergistic relationship.

In summary, prior research suggests that yoga may have beneficial effects on two broad factors that likely influence academic performance: cognitive abilities (including attention and learning) and psychosocial well-being. We predict that yoga may exert these effects via self-regulatory processes that mitigate stress [28]. However, at the moment, potential causal relationships among these factors are unclear. While psychosocial outcome measures were collected as part of the original study, the study was not designed or powered to test the potential relationships among yoga participation, psychosocial outcomes, and GPA. Given that prior research suggests that self-regulatory strategies may be more influential on GPA than psychosocial factors such as depression [38], it is possible that self-regulation serves as an overarching factor that has downstream influences on stress coping, psychosocial well-being, cognitive abilities, and academic performance. It will be important for future studies to be specifically designed and sufficiently powered to examine these mechanistic hypotheses.

### 5.1. Limitations

This study had several limitations that should be noted. First, the sample size was small and unbalanced. In particular, a larger number of yoga participants were excluded from the analysis compared to controls due to a greater lack of parental consent for obtaining grades data, students dropping out of school, and students opting out of the study, the latter being a possible feasibility issue. A larger sample size may have reduced the possibility of obtaining baseline differences in GPA based on classroom. In addition, the current sample was composed of a rural, predominantly Caucasian demographic, which may limit the generalizability of the findings. However, the average GPAs reported in the present study are similar to the national average GPA in the United States (3.00) [39], suggesting that our results are at least somewhat commensurate with national norms. Finally, previous experience in yoga or mind-body practices was not measured on the demographics questionnaire and would be useful to examine in future studies.

## 6. Conclusions

The present exploratory study suggests that, when compared to PE-as-usual, school-based yoga interventions may have beneficial short-term effects on GPA, particularly while yoga is being practiced regularly. In other words, it may be beneficial for schools to consider implementing yoga classes as part of the standard PE curriculum. However the association between yoga and academic performance warrants subsequent research. A particularly important area for future research relates to the dosage of school-based yoga interventions. The present study incorporated a 12-week yoga intervention; however longitudinal designs are necessary to more definitively evaluate persistence and observe the potential long-term effects of yoga on academic performance. Mechanistic studies are also needed that examine the potential influence of yoga-based changes in self-regulation, stress coping, psychosocial well-being, and cognitive processes (learning and attention) on maintaining or improving academic performance over time.

## Figures and Tables

**Table 1:** Demographic characteristics of participants by group.

	Yoga (*n* = 44)	Control (*n* = 51)
Gender		
Female	50%	63%
Male	50%	37%
Grade		
9th	39%	59%
10th	59%	39%
11th	2%	2%
Race		
White	89%	84%
Asian	2%	2%
More than one race	0%	8%
American Indian	5%	0%
African American	0%	2%
Unknown	5%	4%

**Table 2:** Mean cumulative GPA for each academic quarter by group.

	Yoga (*n* = 44)	Control (*n* = 51)
1st quarter	3.25 ± 0.67	3.24 ± 0.62
2nd quarter	3.00 ± 0.85	3.09 ± 0.72
3rd quarter	3.01 ± 0.51	2.82 ± 0.72
4th quarter	2.68 ± 0.78	2.67 ± 0.80

*Note*. Results are reported as mean ± standard deviation.

## References

[1] Balfanz R., Herzog L., Mac Iver D. J. (2007). Preventing student disengagement and keeping students on the graduation path in urban middle-grades schools: early identification and effective interventions. *Educational Psychologist*.

[2] Durlak J. A., Weissberg R. P., Dymnicki A. B., Taylor R. D., Schellinger K. B. (2011). The impact of enhancing students' social and emotional learning: a meta-analysis of school-based universal interventions. *Child Development*.

[3] Greenberg M. T., Weissberg R. P., O'Brien M. U. (2003). Enhancing school-based prevention and youth development through coordinated social, emotional, and academic learning. *American Psychologist*.

[4] Khalsa S. B. S. (2004). Yoga as a therapeutic intervention: a bibliometric analysis of published research studies. *Indian Journal of Physiology and Pharmacology*.

[5] Butzer B., Day D., Potts A. (2015). Effects of a classroom-based yoga intervention on cortisol and behavior in second- and third-grade students: A pilot study. *Journal of Evidence-Based Complementary and Alternative Medicine*.

[6] Birdee G. S., Yeh G. Y., Wayne P. M., Phillips R. S., Davis R. B., Gardiner P. (2009). Clinical applications of yoga for the pediatric population: a systematic review. *Academic Pediatrics*.

[7] Galantino M. L., Galbavy R., Quinn L. (2008). Therapeutic effects of yoga for children: a systematic review of the literature. *Pediatric Physical Therapy*.

[8] Kaley-Isley L. C., Peterson J., Fischer C., Peterson E. (2010). Yoga as a complementary therapy for children and adolescents: a guide for clinicians. *Psychiatry (Edgemont)*.

[9] Serwacki M. L., Cook-Cottone C. (2012). Yoga in the schools: a systematic review of the literature. *International Journal of Yoga Therapy*.

[10] Mehta S., Mehta V., Mehta S. (2011). Multimodal behavior program for ADHD incorporating yoga and implemented by high school volunteers: a pilot study. *ISRN Pediatrics*.

[11] Nidich S., Mjasiri S., Nidich R. (2011). Academic achievement and transcendental meditation: a study with at-risk urban middle school students. *Education*.

[12] Beauchemin J., Hutchins T. L., Patterson F. (2008). Mindfulness meditation may lessen anxiety, promote social skills, and improve academic performance among adolescents with learning disabilities. *Complementary Health Practice Review*.

[13] Benson H., Wilcher M., Greenberg B. (2000). Academic performance among middle-school students after exposure to a relaxation response curriculum. *Journal of Research and Development in Education*.

[14] Kauts A., Sharma N. (2009). Effect of yoga on academic performance in relation to stress. *International Journal of Yoga*.

[15] Hall P. D. (1999). The effect of meditation on the academic performance of african american college students. *Journal of Black Studies*.

[16] Pontifex M. B., Hillman C., McAuley E. (2013). The acute effects of yoga on executive function neha gothe1. *Journal of Physical Activity and Health*.

[17] Rosaen C., Benn R. (2006). The experience of transcendental meditation in middle school students: a qualitative report. *Explore: The Journal of Science and Healing*.

[18] Sibinga E. M. S., Kerrigan D., Stewart M., Johnson K., Magyari T., Ellen J. M. (2011). Mindfulness-based stress reduction for urban youth. *The Journal of Alternative and Complementary Medicine*.

[19] Conboy L. A., Noggle J. J., Frey J. L., Kudesia R. S., Khalsa S. B. S. (2013). Qualitative evaluation of a high school yoga program: feasibility and perceived benefits. *Explore*.

[20] Kuncel N. R., Credé M., Thomas L. L. (2005). The validity of self-reported grade point averages, class ranks, and test scores: a meta-analysis and review of the literature. *Review of Educational Research*.

[21] Sharma M. (2014). Yoga as an alternative and complementary approach for stress management: a systematic review. *Journal of Evidence-Based Complementary and Alternative Medicine*.

[22] Davidson R. J., Dunne J., Eccles J. S. (2012). Contemplative practices and mental training: prospects for american education. *Child Development Perspectives*.

[23] Massachusetts Department of Elementary and Secondary Education (ESE) Enrollment data (2009-10)—monument mt regional high. http://profiles.doe.mass.edu/profiles/student.aspx?orgcode=06180505&orgtypecode=6&&fycode=2010.

[24] Micceri T. (1989). The Unicorn, The Normal Curve, and Other Improbable Creatures. *Psychological Bulletin*.

[25] Greenberg M. T., Harris A. R. (2012). Nurturing mindfulness in children and youth: current state of research. *Child Development Perspectives*.

[26] Grove W. A., Wasserman T. (2004). The life-cycle pattern of collegiate GPA: longitudinal cohort analysis and grade inflation. *The Journal of Economic Education*.

[27] Trudeau F., Shephard R. J. (2008). Physical education, school physical activity, school sports and academic performance. *International Journal of Behavioral Nutrition and Physical Activity*.

[28] Gard T., Noggle J. J., Park C. L., Vago D. R., Wilson A. (2014). Potential self-regulatory mechanisms of yoga for psychological health. *Frontiers in Human Neuroscience*.

[29] Sprengel M., Fritts M. (2012). OA13.02. Utilizing mind-body practices in public schools: teaching self-regulation skills and fostering resilience in our next generation. *BMC Complementary and Alternative Medicine*.

[30] Hedges D. W., Woon F. L. (2011). Early-life stress and cognitive outcome. *Psychopharmacology*.

[31] Manjunath N. K., Telles S. (2001). Improved performance in the tower of london test following yoga. *Indian Journal of Physiology and Pharmacology*.

[32] Peck H. L., Kehle T. J., Bray M. A., Theodore L. A. (2005). Yoga as an intervention for children with attention problems. *School Psychology Review*.

[33] Flook L., Smalley S. L., Kitil M. J. (2010). Effects of mindful awareness practices on executive functions in elementary school children. *Journal of Applied School Psychology*.

[34] Fröjd S. A., Nissinen E. S., Pelkonen M. U. I., Marttunen M. J., Koivisto A.-M., Kaltiala-Heino R. (2008). Depression and school performance in middle adolescent boys and girls. *Journal of Adolescence*.

[35] Quiroga C. V., Janosz M., Bisset S., Morin A. J. S. (2013). Early adolescent depression symptoms and school dropout: mediating processes involving self-reported academic competence and achievement. *Journal of Educational Psychology*.

[36] Khalsa S. B. S., Hickey-Schultz L., Cohen D., Steiner N., Cope S. (2012). Evaluation of the mental health benefits of yoga in a secondary school: a preliminary randomized controlled trial. *Journal of Behavioral Health Services and Research*.

[37] Noggle J. J., Steiner N. J., Minami T., Khalsa S. B. S. (2012). Benefits of yoga for psychosocial well-being in a us high school curriculum: a preliminary randomized controlled trial. *Journal of Developmental and Behavioral Pediatrics*.

[38] Richardson M., Abraham C., Bond R. (2012). Psychological correlates of university students' academic performance: a systematic review and meta-analysis. *Psychological Bulletin*.

[39] Nord C., Roey S., Perkins R. (2011). The nation's report card: America's high school graduates. *NCES*.

